# An exonic insertion in the *NAGLU* gene causing Mucopolysaccharidosis IIIB in Schipperke dogs

**DOI:** 10.1038/s41598-020-60121-3

**Published:** 2020-02-21

**Authors:** Karthik Raj, N. Matthew Ellinwood, Urs Giger

**Affiliations:** 1 Section of Medical Genetics (PennGen), School of Veterinary Medicine, University of Pennsylvania, Philadelphia, PA 19104-6010 USA; 2 Present Address: College of Agriculture and Life Sciences, Iowa State University, Ames, Iowa USA

**Keywords:** Animal breeding, Clinical genetics, Genetic association study, Genomics, Genotype, Mutation, Sequencing, Genetics, Molecular biology, Diseases, Metabolic disorders

## Abstract

Mucopolysaccharidosis (MPS) IIIB (Sanfilippo syndrome B; OMIM 252920), is a lysosomal storage disease with progressive neurological signs caused by deficient activity of alpha-N-acetylglucosaminidase (NAGLU, EC 3.2.1.50). Herein we report the causative variant in the *NAGLU* gene in Schipperke dogs and a genotyping survey in the breed. All six exons and adjacent regions of the *NAGLU* gene were sequenced from six healthy appearing and three affected Schipperkes. DNA fragment length and TaqMan assays were used to genotype privately owned Schipperkes. A single variant was found in exon 6 of MPS IIIB affected Schipperkes: an insertion consisting of a 40–70 bp poly-A and an 11 bp duplication of the exonic region preceding the poly-A (XM_548088.6:c.2110_2111ins[A(40_70);2100_2110]) is predicted to insert a stretch of 13 or more lysines followed by either an in-frame insertion of a repeat of the four amino acids preceding the lysines, or a frameshift. The clinically affected Schipperkes were homozygous for this insertion, and the sequenced healthy dogs were either heterozygous or homozygous for the wild-type allele. From 2003–2019, 3219 Schipperkes were genotyped. Of these, 1.5% were homozygous for this insertion and found to be clinically affected, and 23.6% were heterozygous for the insertion and were clinically healthy, the remaining 74.9% were homozygous for the wild-type and were also clinically healthy. The number of dogs homozygous and heterozygous for the insertion declined rapidly after the initial years of genotyping, documenting the benefit of a DNA screening program in a breed with a small gene pool. In conclusion, a causative *NAGLU* variant in Schipperke dogs with MPS IIIB was identified and was found at high frequency in the breed. Through genotyping and informed breeding practices, the prevalence of canine MPS IIIB has been drastically reduced in the Schipperke population worldwide.

## Introduction

The mucopolysaccharidoses (MPS) are a group of hereditary lysosomal storage disorders in which specific glycosaminoglycans accumulate in lysosomes due to various enzyme deficiencies. There are up to 12 individual MPS types described in humans^1^ and animals^2^ with all but MPS II showing autosomal recessive inheritance. Genetic variants in dogs have been identified in the genes associated with MPS I^3^, IIIA^4,5^, VI^6,7^, and VII^8,9^. Clinical manifestations of MPS in dogs are most commonly ocular and musculoskeletal^2^. In contrast, MPS III, also known as Sanfilippo Syndrome, causes a progressive and primarily neurological disease^4,5,10^. At the cellular level it is characterized by primary lysosomal accumulation of heparan sulfate and secondary lysosomal storage of gangliosides^1^.

Mucopolysaccharidosis IIIB (also known as Sanfilippo syndrome B) is caused by variants in the alpha-N-acetylglucosaminidase (*NAGLU*) gene and has been previously characterized at the clinical and molecular level in humans, emus^11^, cattle^12^, knockout mice^13^, and transgenic swine^14,15^. Furthermore, MPS IIIB has been clinicopathologically reported in Schipperke dogs (OMIA 001342-9615)^10^. At approximately two years of age, affected Schipperkes develop a slowly progressive ataxia leading to humane euthanasia before six years of age^10^. This study documents the causative *NAGLU* gene variant in Schipperke dogs with MPS IIIB and also demonstrates the high initial frequency of this allele in the breed, and its reduction after inauguration of genotype screening for this variant allele in the breed.

## Methods

Ethylenediaminetetraacetic acid (EDTA) anticoagulated blood and cheek swab/brush samples were sent for the diagnosis of MPS IIIB or genotyping to PennGen Laboratories at the University of Pennsylvania, Philadelphia, Pennsylvania USA from clinically affected and healthy-appearing Schipperke dogs. Affected status was based on the reported onset of severe and progressive ataxia with onset at two to three years of age based on communication from owners or referring veterinarians. The studies were approved by the Institutional Animal Care and Use Committee (IACUC) of the University of Pennsylvania.

Briefly, DNA was extracted from EDTA blood and cheek swab/brush samples using the QIAamp DNA Blood Mini Kit (Qiagen, Hilden, Germany). The six exons of the *NAGLU* gene were sequenced from nine Schipperkes including six healthy appearing and three clinically affected with MPS IIIB. Primers were designed, based on the published canine reference sequences (CanFam3.1 and XM_548088.6) to cover all six exons and at least 90 bp of intronic sequence adjacent to the exons (Table 1). The PCR-amplified products (amplified using KOD Xtreme Hot Start DNA Polymerase, EMD Millipore Corp., Billerica, MA, USA) were submitted for direct Sanger sequencing at the University of Pennsylvania’s Sequencing Core Facility. The DNA sequences were then aligned to dog reference sequences (CanFam3.1 and XM_548088.6) to find genetic variants in the exons and splice-sites of the *NAGLU* gene.

**Table 1:** Primers used for amplifying and Sanger sequencing exonic and flanking regions of canine *NAGLU* gene and for genotyping the causative insertion and wild-type alleles in Schipperkes.

Exon #	Exon length (bp)	Forward Primer	Reverse Primer	Amplicon Length (bp)	Annealing Temperature (°C)
1	424	ATGTGAAAGCTCTCCAGGTACA	CGATGTCACCGTTTCCATTCTTC	925	66
2	148	GTGAGTCCTGGAGTGAAACAGT	TAGCGTTTCTAGTGAGGTGCTG	485	65
3–4	147 (Exon 3) 86 (Exon 4)	TTGCAACAAAGCTGACCCATTAG	GCTGCCATTTGCTAAGACTGTG	798	67
5	257	CACTGCTCCATCTAGGACTCTG	AGTGCTTGGTCAACTGTCAAGG	682	67
6a	1450	GACAACACTGCCCTAGAGATCC	CCTCGCCTCCACATAGTACAAG	953	67
6b		ATGGTTACCACTGTCTGGTACA	AAACGTATTGGGAGAGGATTCCC	1043	65
Genotyping		GCATTCCCTTCCAACAGCACCAGT	GCCCACAAGGAGCCAGCCACCAAT	169	68

Primer pairs 6a and 6b were designed to amplify and sequence exon 6 to make it across this relatively long exon.

Sanger sequencing was unable to accurately determine the exact sequence of the long homopolymer poly-A insertion in the affected dogs (data not shown). Consequently, the insert sizes were estimated based upon either gel separation or Sanger sequencing. In addition, for genotyping, a specific primer pair (Table 1) was designed to amplify the region surrounding the exon 6 insertion, with subsequent PCR products subjected to fragment length analysis using electrophoresis on a 6% polyacrylamide gel. The fragments’ size, either consistent with the mutant and/or wild-type alleles, was the basis for genotyping. To control for any potential allelic dropout issues, samples were separately tested twice. A TaqMan genotyping assay was subsequently developed: briefly, a VIC dye-labelled probe (TGACAAGAATGCCTTCCAGCT) was designed to anneal to the insertion site of wild-type allele and a FAM dye-labelled probe (CAAGAATGCCTTCCAAAAA) to a unique sequence of the variant allele with the insertion and the primers used for the amplification are CTGGGTGCCGAAGATAAAGGT and CCCTTCCAACAGCACCAGTT. Genotyping data for MPS IIIB in the Schipperke pet population based on samples submitted to the PennGen Laboratories from 2003 to 2019 was gathered and analyzed.

## Results

**Figure 1:**
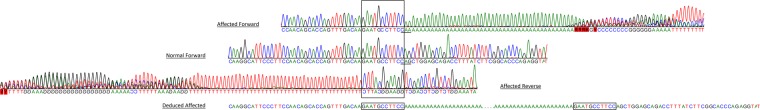
Sanger sequencing chromatograms of a region in exon 6 of *NAGLU* gene from a Schipperke with MPS IIIB (forward and reverse) compared to the normal canine sequence (forward). The affected reverse sequence is flipped horizontally to align to the normal. The sequence traces show the poly-A/poly-T and loss of sequence quality, while trying to make it past the poly-A/poly-T insertion. An 11 bp region (boxed) is seen in both the forward and reverse sequences before the poly-A and poly-T respectively, demonstrating a duplication. The 704^th^ codon in the normal forward (CAG) and in the affected forward (CAA) at the junction of the insertion are underlined, it codes for glutamine in both (a synonymous variant). The affected sequence flanking the insertion deduced from the forward and reverse sequences is shown at the bottom of the figure.

When aligning Sanger sequencing data of the exonic and surrounding intronic regions of the *NAGLU* gene from clinically healthy Schipperkes and those Schipperkes affected with MPS IIIB to the canine reference sequences (CanFam3.1 and XM_548088.6), only one single variant was discovered. The sixth and last exon of the *NAGLU* gene contains an insertion (XM_548088.6:c.2110_2111ins[A(40_70);2100_2110]) comprised of a homopolymer of A residues (poly-A) and an 11 bp duplication of the sequence directly upstream of the poly-A. Three clinically affected dogs were found to be homozygous for this insertion, four clinically healthy dogs were heterozygous, and two clinically healthy dogs were homozygous for the wild-type allele. The poly-A region was at least 40 bp in length, however the exact length of the poly-A in the insert could not be determined from the Sanger sequencing data as the sequencing read quality decreased, presumably due to variation between the two allele insert sizes, and/or due to “slippage” of the polymerases during amplification or sequencing (Fig. 1).

**Figure 2:**
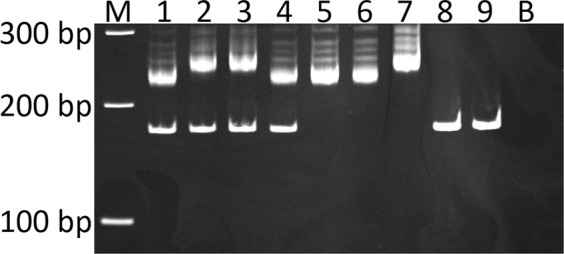
Polyacrylamide gel (6%) electrophoresis of the amplified fragments to detect the *NAGLU* insertion in Schipperkes with and without MPS IIIB. M is 100 bp marker, 1–4 heterozygotes, 5–7 are homozygous dogs for the insertion, 8 and 9 homozygous wild-type/normal, and B blank/negative control. Notable is the considerable variation in insert size in different individuals. This is a cropped gel image.

The primer pair designed to be used for genotyping by gel electrophoresis amplified a wild-type fragment predicted to be 169 bp and a longer fragment for the variant allele with the insertion (Fig. 2). The fragments with the insertion were at least 50 bp longer than the wild-type fragment, but the insert lengths varied markedly between individual Schipperkes, ranging between 50–80 bp.

**Figure 3:**
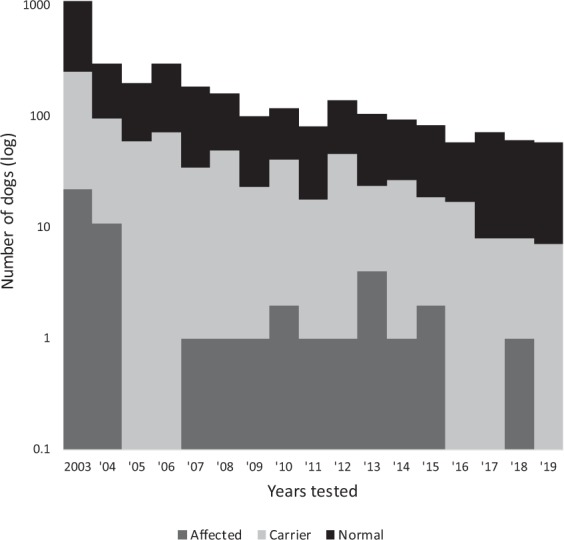
Survey of Schipperkes for the exonic insertion in *NAGLU* gene from 2003–2019. Note this is a semi-log chart.

From 2003 to 2019, a total of 3,219 Schipperkes were genotyped at PennGen Laboratories. Of the total number genotyped 2,411 (74.9%) Schipperkes were homozygous for the wild-type allele, 760 (23.6%) were heterozygous, and 48 (1.5%) were homozygous for the insertion (Table 2 and Fig. 3). All Schipperkes homozygous for the variant had or developed clinical signs of MPS IIIB unless lost to follow up before reaching the age of onset of clinical signs (≥2 years). As this was a genotyping survey, there was no means to follow cases closely. Of the 48 animals tested as homozygous for the mutation, 54.2% (n = 26) were of an age where they were definitively displaying clinical signs of MPS IIIB. The remaining animals were younger than the extreme limit for onset of signs (3 years of age). Of these dogs (n = 22), none were subsequently reported as not developing disease, but may well have been euthanized before the expected age of disease onset.

**Table 2:** Genotyping results of Schipperkes for the exonic insertion in *NAGLU* gene from 2003–2019.

Genotype	All Dogs	Gender			Year Tested																
		Female	Male	NA*	2003	2004	2005	2006	2007	2008	2009	2010	2011	2012	2013	2014	2015	2016	2017	2018	2019
Affected	48	12	28	8	22	11	0	0	1	1	1	2	1	1	4	1	2	0	0	1	0
Carrier	760	391	343	26	234	86	60	72	34	49	22	39	17	45	20	26	17	17	8	7	7
Normal	2411	1290	1092	29	841	200	142	228	153	111	78	77	64	93	81	67	65	42	64	53	52
Total	3219	1693	1463	63	1097	297	202	300	188	161	101	118	82	139	105	94	84	59	72	61	59

*NA is not available.

The number of Schipperke samples submitted for genotyping rapidly and drastically declined from the first years of screening. Similarly, the number of samples from Schipperkes that were genotyped as homo- and heterozygous for the insertion declined. However, while in absolute numbers, the mutant allele numbers decreased strikingly, the frequency of the allele in submitted samples did not decrease per year, and there have even been recent carriers and (rarely) affected dogs identified (Table 2 and Fig. 3). Screening of Schipperkes from North America, Europe, Australasia, and Russia revealed carrier dogs in all these regions, indicating the worldwide distribution of the mutant allele (data not shown).

## Discussion

The canine *NAGLU* gene is on chromosome 9 and comprised of six exons (XM_548088.6) with exon 6 being by far the longest (1450 bp). It codes for the lysosomal acid hydrolase alpha-N-acetylglucosaminidase (EC 3.2.1.50) which consists of 747 amino acids (XP_548088.2), including the signal sequence. The exonic *NAGLU* sequence from all nine dogs sequenced was identical to the reference genomic sequence except for the disease-associated variant. The protein sequence shows close homology to human (86% identity) and other mammalian (76–90%) sequences which is expected for a housekeeping gene (https://www.ncbi.nlm.nih.gov/homologene/222).

Schipperkes with MPS IIIB have a *NAGLU* insertion near the end of exon 6, which contains a poly-A insertion followed by a duplication of the preceding 11 bp of wild-type sequence. The sequence of this 11 base pair repeat in the native context is flanked by AA (two adenines) at the 5′ end and A (one adenine) at the 3′ end. Since the insertion is a poly-A sequence, and the molecular mechanism of the sequence repetition is not known, it could actually have been a 14 bp repeat of the native sequence. We have chosen the conservative assessment that the A residues at both ends of the insertion were part of an exogenous poly-A insert. The insertion is predicted to result in the addition of many lysines after the 704^th^ amino acid (a glutamine that results from a synonymous variant caused by the insertion) in the NAGLU protein with three potential consequences past the stretch of inserted lysines depending on the actual length of the poly-A insertion: (1) It stays in-frame with an insertion of a repeat of the four native amino acids preceding the lysines (asparagine, alanine, phenylalanine, glutamine), or (2) it causes a frameshift with an early stop-codon, or (3) frameshift with the lack of a stop-codon. In any case, the exonic insertion is predicted to disrupt the C-terminal end of the enzyme in affected dogs. We had shown the lack of NAGLU enzyme activity and lysosomal storage in affected dogs, but neither immunoblotting nor gene expression studies were performed to further confirm the disruptive nature of this genetic variant.

A review of the human *NAGLU* gene sequence in ClinVar (https://www.ncbi.nlm.nih.gov/clinvar) accessed on (October 14, 2019) contains 181 variants. Of the 77 that are labelled as pathogenic or likely pathogenic, 42 are in exon 6. However, only one (c.2116C>T, p.Gln706Ter) is near the location of the insertion seen in MPS IIIB Schipperkes. The c.2116C>T variant was reported in a 6 year old female child with severe degenerative neuropathy due to MPS IIIB^16^.

Interestingly, there are several disease-causing poly-A insertions known in dogs that have the same pattern of a poly-A flanked by a duplicated/repeated native sequence at both ends^17–21^. Such inserts with characteristic repeats may likely be the result of a target primed reverse transcription mechanism^22^. Some are also known to exhibit varied length of their poly-A, for example, the *FXI* variant in the Kerry Blue Terriers with Factor XI deficiency^17^. In cattle and emus with MPS IIIB, the disease-causing *NAGLU* variants are a missense (c.1354G>A, p.Glu452Lys) and frameshift deletion (c.1098_1099delGG), respectively, and both are also located in exon 6.

Occasionally when genotyping heterozygotes by fragment length, the amplification preferentially produced the smaller wild-type amplicon and failed to amplify the larger fragment, resulting in allelic dropout in heterozygotes. This did not appear to be a factor in the homozygous affected dogs. This preferential amplification in rare circumstances could have led to misidentification of heterozygotes as homozygotes for the wild-type allele. A cause was not identified and analyzing the samples in separate assays eliminated the allelic dropout issue. The TaqMan genotyping assay clearly discriminated all three genotypes, and this technique was not affected by any apparent allelic dropout artifact.

Based upon the devastating progressive clinical course of canine MPS IIIB in Schipperkes, breeders, owners, and veterinary clinicians were eager to genotype their dogs and patients. And while this represents a biased population within the breed, a striking number of homozygous and heterozygous dogs for the mutant allele were identified. The survey of *NAGLU* variant genotyping results in Schipperkes shows characteristic dynamics for a canine breed with a small gene pool. According to the UK Kennel Club only ≤51 (https://www.thekennelclub.org.uk/media/129029/10yrstatsutility.pdf) puppies were registered each year, from 2009 to 2018. Before the disease was discovered at the turn of the century and the molecular basis was established, no clinical screening test for MPS IIIB carrier dogs was available, and the prevalence of the mutant allele was not known. Following the initiation of genotyping in 2003, about one third of 3,219 Schipperkes whose genotypes are presented herein were tested in the first year of screening and another one third during the following five years. The last third of dogs were genotyped during next 11 years with <140 dogs tested per year. Strikingly, in the first year of genotyping, the heterozygotes represented 21.3% of the total of 1,097 animals screened that year. Overall, dogs homozygous and heterozygous for the mutant allele were 1.5% and 23.6%, respectively, indicating a mutant allele frequency of 0.133 which is high for a canine breed and reflects an ancient popular sire/dam effect, a population bottleneck, and/or close inbreeding affecting the Hardy-Weinberg equilibrium. Indeed a potential founder individual up to eight generations deep was found in the pedigree, with six separate lines of descent, which was further compounded by multiple lines of decent from two intermediate animals^10^. While a specific popular sire/dam was not identified through testing, the mutant allele was widespread in the breeding population worldwide.

Our genotyping was in complete concordance with phenotype, except for those dogs too young (<3 years) to show clinical signs and lost to follow up. There is no evidence that heterozygous Schipperkes have any advantage over homozygous wild-type animals, but likely the lines of dogs with the mutant allele had other desirable traits to be used for breeding worldwide. Also during the first year of genotyping, many more affected and carrier dogs were found than in subsequent years, reflecting the impact of the genotyping program. As this is a storage disease with an adult onset of clinical signs, it was noted that some affected dogs were in the breeding pool. Our recommendation for screening was initially to test all breeding Schipperkes and to perform only two types of matings: (1) matings between dogs free of the mutant allele or (2) matings of carrier dogs with proven clear dogs. While we recommended direct testing of all breeding dogs, it was likely that breeders used the initial results for subsequent breeding selection and assumed they were clear by descent. The rare affected dogs were likely the result of parents that were not tested or mis-parentage. The marked reduction in Schipperkes homozygous or heterozygous for the variant indicates the positive impact the screening had on the breeding population. While PennGen was the only diagnostic laboratory offering genotyping, the survey is still biased by breeder and pet owner interest, the discovery of carrier and affected dogs in certain breeding lines and kennels, as well as the further use of carrier dogs for breeding. Once all breeding dogs are screened and parentage is assured, there may thereafter no longer be a need for screening. Similarly, beneficial effects by genotype screening were seen with various other serious hereditary disease traits in specific canine breeds including copper toxicosis^23^, leukocyte adhesion deficiency^24^, and myotonia congenita^25^.

In conclusion, a causative variant of MPS IIIB in Schipperke dogs was identified, found to be widely disseminated in the breed, and drastically reduced in the Schipperke population worldwide by effective genotyping and breeding practices.

## Supplementary information

Supplementary information.

## Data Availability

All data is made available either in the manuscript or as Supplementary Information. Any data generated and/or analyzed during this study and if not already included in the manuscript or Supplementary Information will be made available from the corresponding author on reasonable request.

## References

[1] Neufeld, E. F. & Muenzer, J. The Mucopolysaccharidoses in The Online Metabolic and Molecular Bases of Inherited Disease (eds Valle, D., Antonarakis, S., Ballabio, A., Beaudet, A. & Mitchell, G. A.), http://ommbid.mhmedical.com/content.aspx?bookid=2709&sectionid=225544161. (McGraw-Hill, New York, Accessed October 25, 2019).

[2] Haskins, M. & Giger, U. Chapter 24 - Lysosomal Storage Diseases. Clinical Biochemistry of Domestic Animals (Sixth Edition) Sixth Edition, 731–749 (2008).

[3] Menon KP, Tieu PT, Neufeld EF (1992). Architecture of the canine IDUA gene and mutation underlying canine mucopolysaccharidosis I. Genomics.

[4] Aronovich EL (2000). Canine Heparan Sulfate Sulfamidase and the Molecular Pathology Underlying Sanfilippo Syndrome Type A in Dachshunds. Genomics.

[5] Yogalingam G, Pollard T, Gliddon B, Jolly RD, Hopwood JJ (2002). Identification of a Mutation Causing Mucopolysaccharidosis Type IIIA in New Zealand Huntaway Dogs. Genomics.

[6] Jolly R (2012). Mucopolysaccharidosis type VI in a Miniature Poodle-type dog caused by a deletion in the arylsulphatase B gene. New Zealand Veterinary Journal.

[7] Wang P (2018). Mucopolysaccharidosis Type VI in a Great Dane Caused by a Nonsense Mutation in the ARSB Gene. Veterinary Pathology.

[8] Silverstein Dombrowski DC (2004). Mucopolysaccharidosis type VII in a German Shepherd Dog. Journal of the American Veterinary Medical Association.

[9] Hytönen MK (2012). A Novel GUSB Mutation in Brazilian Terriers with Severe Skeletal Abnormalities Defines the Disease as Mucopolysaccharidosis VII. PloS one.

[10] Ellinwood N (2003). A model of mucopolysaccharidosis IIIB (Sanfilippo syndrome type IIIB): N-acetyl-α-D-glucosaminidase deficiency in Schipperke dogs. J Inherit Metab Dis.

[11] Aronovich EL, Johnston JM, Wang P, Giger U, Whitley CB (2001). Molecular Basis of Mucopolysaccharidosis Type IIIB in Emu (Dromaius novaehollandiae): An Avian Model of Sanfilippo Syndrome Type B. Genomics.

[12] Karageorgos L, Hill B, Bawden MJ, Hopwood JJ (2007). Bovine mucopolysaccharidosis type IIIB. J Inherit Metab Dis.

[13] Li HH (1999). Mouse Model of Sanfilippo Syndrome Type B Produced by Targeted Disruption of the Gene Encoding α -N-acetylglucosaminidase. Proceedings of the National Academy of Sciences of the United States of America.

[14] Zhao X (2016). Production of Transgenic Pigs with an Introduced Missense Mutation of the Bone Morphogenetic Protein Receptor Type IB Gene Related to Prolificacy. Asian-Australasian journal of animal sciences.

[15] Yang Q (2018). A model of mucopolysaccharidosis type IIIB in pigs. Biology open.

[16] Zhao HG, Aronovich EL, Whitley CB (1998). Genotype-Phenotype Correspondence in Sanfilippo Syndrome Type B. The American Journal of Human Genetics.

[17] Tcherneva E, Giger U (2007). Molecular base of coagulation factor XI deficiency in Kerry Blue Terrier. Bulgarian Journal of Veterinary Medicine.

[18] Bishop MA, Xenoulis PG, Levinski MD, Suchodolski JS, Steiner JM (2010). Identification of variants of the SPINK1 gene and their association with pancreatitis in Miniature Schnauzers. American journal of veterinary research.

[19] Miyadera K, Brierley I, Aguirre-Hernández J, Mellersh CS, Sargan DR (2012). Multiple Mechanisms Contribute to Leakiness of a Frameshift Mutation in Canine Cone-Rod Dystrophy. PloS one.

[20] Turba ME, Loechel R, Rombolà E, Gandini G, Gentilini F (2017). Evidence of a genomic insertion in intron 2 of SOD1 causing allelic drop-out during routine diagnostic testing for canine degenerative myelopathy. Animal Genetics.

[21] Lit L, Belanger JM, Boehm D, Lybarger N, Oberbauer AM (2013). Differences in Behavior and Activity Associated with a Poly(A) Expansion in the Dopamine Transporter in Belgian Malinois. PloS one.

[22] Ostertag EM, Kazazian HH (2001). Biology of mammalian L1 retrotransposons. Annual Review of Genetics.

[23] Fieten H (2016). The Menkes and Wilson disease genes counteract in copper toxicosis in Labrador retrievers: a new canine model for copper-metabolism disorders. Disease models & mechanisms.

[24] Kijas JMH (1999). A Missense Mutation in the β-2 Integrin Gene (ITGB2) Causes Canine Leukocyte Adhesion Deficiency. Genomics.

[25] Bhalerao DP, Rajpurohit Y, Vite CH, Giger U (2002). Detection of a genetic mutation for myotonia congenita among Miniature Schnauzers and identification of a common carrier ancestor. American journal of veterinary research.

